# CUX1 attenuates the apoptosis of renal tubular epithelial cells induced by contrast media through activating the PI3K/AKT signaling pathway

**DOI:** 10.1186/s12882-024-03625-8

**Published:** 2024-06-07

**Authors:** Rong-Zheng Yue, Jing Wang, Feng Lin, Cong-Jun Li, Bai-Hai Su, Rui Zeng

**Affiliations:** 1 Department of Nephrology, Kindey Research Institute, West China Hospital of Sichuan University, Chengdu, Sichuan 610041 China; 2 Department of Cardiovascular diseases, West China Hospital, School of Clinic Medicine, Sichuan University, Chengdu, Sichuan 610041 China

**Keywords:** Renal tubular epithelial cells, Contrast media (CM), PI3K/AKT signaling pathway, CUT-like homeobox 1 (CUX1), Contrast-induced acute kidney injury

## Abstract

**Objective:**

Contrast media (CM) is a commonly applied drug in medical examination and surgery. However, contrast-induced acute kidney injury (CIAKI) poses a severe threat to human life and health. Notably, the CUT-like homeobox 1 (CUX1) gene shows protective effects in a variety of cells. Therefore, the objective of this study was to provide a new target for the treatment of CIAKI through exploring the role and possible molecular mechanism of CUX1 in CIAKI.

**Method:**

Blood samples were collected from 20 patients with CIAKI and healthy volunteers. Human kidney 2 (HK-2) cells were incubated with 200 mg/mL iohexol for 6 h to establish a contrast-induced injury model of HK-2 cells. Subsequently, qRT-PCR was used to detect the relative mRNA expression of CUX1; CCK-8 and flow cytometry to assess the proliferation and apoptosis of HK-2 cells; the levels of IL(interleukin)-1β, tumor necrosis factor alpha (TNF-α) and malondialdehyde (MDA) in cells and lactate dehydrogenase (LDH) activity in cell culture supernatant were detect; and western blot to observe the expression levels of CUX1 and the PI3K/AKT signaling pathway related proteins [phosphorylated phosphoinositide 3-kinase (p-PI3K), PI3K, phosphorylated Akt (p-AKT), AKT].

**Results:**

CUX1 expression was significantly downregulated in blood samples of patients with CIAKI and contrast-induced HK-2 cells. Contrast media (CM; iohexol) treatment significantly reduced the proliferation of HK-2 cells, promoted apoptosis, stimulated inflammation and oxidative stress that caused cell damage. CUX1 overexpression alleviated cell damage by significantly improving the proliferation level of HK-2 cells induced by CM, inhibiting cell apoptosis, and reducing the level of LDH in culture supernatant and the expression of IL-1β, TNF-α and MDA in cells. CM treatment significantly inhibited the activity of PI3K/AKT signaling pathway activity. Nevertheless, up-regulating CUX1 could activate the PI3K/AKT signaling pathway activity in HK-2 cells induced by CM.

**Conclusion:**

CUX1 promotes cell proliferation, inhibits apoptosis, and reduces inflammation and oxidative stress in CM-induced HK-2 cells to alleviate CM-induced damage. The mechanism of CUX1 may be correlated with activation of the PI3K/AKT signaling pathway.

**Supplementary Information:**

The online version contains supplementary material available at 10.1186/s12882-024-03625-8.

## Introduction

Acute kidney injury (AKI) is a common clinical syndrome characterized by abnormal renal function and structure and has become a worldwide public health problem [1]. Contrast-induced acute kidney injury (CIAKI) occurs in up to 30% of patients who receive iodinated contrast media (CM) and is considered to be the third most common cause of hospital-acquired AKI [2]. The diagnostic demand required for various diseases means that patients must undergo interventional radiology, coronary artery examination, and interventional surgery. However, with the increasing number of these examinations or medical treatments, the incidence of CIAKI continues to rise [3]. The increase in the number of patients with CIAKI has been identified as being closely related to the increase in hospitalization time, medical expenses, and long-term mortality [4]. AKI is mainly manifested by a relative increase (25%) or an absolute increase (0.5 mg/dL) in serum creatinine (SCr) [5]. CIAKI has been shown to occur in as many as 10% patients with normal kidney function. Furthermore, the incidence of CIAKI may increase by up to 25% in patients with kidney dysfunction or diabetes, elderly patients, and patients taking nephrotoxic medications and is associated with higher mortality in these patients [6]. Although the mechanism of CIAKI is not fully understood, most studies have concluded that CM mainly activates renal tubular epithelial cell apoptosis through directly inducing toxicity, vasoconstriction, and oxidative stress, thereby causing loss of kidney function and AKI [7]. Currently, better clinical treatment strategies are unavailable to replace kidney replacement therapy [8]. The diagnosis of CIAKI depends on the change in the level of SCr. However, as an indirect and insensitive index during the acute change of kidney function, SCr cannot meet the requirements of accurate diagnosis of CIAKI [9]. Biomarker-based assessments of AKI severity or progression may help predict prognosis and establish treatment directions for individual patients [1]. Therefore, new diagnostic and therapeutic targets are urgently required.

CUT-like homeobox 1 (CUX1) belongs to the homeodomain transcription factor family, which was previously called CDP (CCAAT displacement protein) or CUT-like 1 (CUTL1) [10]. CUX1 can bind the CCAAT promoter element and other transcriptional activators. Moreover, CUX1 is considered both to inhibit and activate transcription [11, 12]. CUX1 is reportedly involved in the regulation of numerous physiological activities, including tissue development, cell proliferation, differentiation and migration, and DNA damage [13]. More interestingly, CUX1 was found to have dual effects of promoting or inhibiting tumors [10]. Sharma et al. demonstrated that the expression of CUX1 is particularly important in the development of kidney as CUX1 ensures the normal development of pretubular aggregates and capillary loop stage glomeruli [14]. However, a subsequent study revealed the positive correlation between the expression level of CUX1 and the development of polycystic kidney disease [15]. Although the function of CUX1 in CIAKI has not been reported, related research has confirmed that CUX1 can activate the phosphatidylinositol 3 kinase (PI3K)/protein kinase B (AKT) pathway [16], which is usually inhibited in CIAKI [17]. Overall, CUX1 may play a key role in the development of CIAKI. Therefore, based on the effect of CUX1 on the PI3K/AKT pathway, we explored and clarified the role and mechanism of CUX1 in CIAKI to identify new biomarkers and therapeutic targets for the diagnosis of CIAKI.

## Data and methods

### Collection of clinical samples

Blood samples were collected from patients with CIAKI (CIAKI group) admitted to West China Hospital, Sichuan University and from healthy volunteers (healthy group) from January 2020 to January 2023. The study procedures, including the blood sampling, were thoroughly explained to all participants. Before participation, each subject was provided with a consent form, which they read and signed to confirm their informed consent. This study was approved by Ethics Committee of West China Hospital, Sichuan University. Inclusion criteria were: (1) Scr was increased by 25% at least from the baseline value; (2) the content of Scr was increased by 0.5 mg/dL; (3) the change in Scr content occurred 48–72 h after the administration of CM. Exclusion criteria were: (1) patients who suffered from other kidney diseases aside from the damage caused by CM; (2) patients who were diagnosed with any infectious disease, inflammation, or immune disease in the past 3 months [18].

### Cell culture and grouping

Renal proximal tubular epithelial cells (HK-2; SCSP-511) were purchased from National Collection of Authenticated Cell Cultures. HK-2 cells were cultured in Dulbecco’s modified Eagle medium (DMEM) containing 10% fetal bovine serum (FBS) and 1% penicillin/streptomycin and cultured in an incubator with 5% CO_2_ at 37 °C.

A CIAKI model was established by treating HK-2 cells with 200 mg/mL iohexol (Shanghai Xu-dongHaipu Pharmaceutical Co., Ltd., China) for 6 h as previously described [19]. When reaching 80% confluency, cells were digested with trypsin and seeded into a 6-well plate. Normally cultured HK-2 cells were set as control group. Besides, HK-2 cells were incubated with 200 mg/mL CM (iohexol, Shanghai Xu-dongHaipu Pharmaceutical Co., Ltd., China) for 6 h to construct a CIAKI model (CM group). In the CM + vector and CM + CUX1 groups, CM-induced HK-2 cells were separately transfected (lipofectamine 2000) with negative pcDNA3.1 vector and CUX1 over-expression vector (pcDNA3.1-CUX1). HK-2 cells were cultured in an incubator for 48 h for subsequent trials.

### Real-time-quantitative PCR (RT-qPCR)

An RNA extraction kit (RC112-01, Vazyme, Nanjing, China) was used to extra RNA from blood samples and at least 1 × 10^7^ cells; a NanoDrop (840-317400, ThermoFisher, USA) to measure the RNA concentration. Next, a HiScript II Reverse Transcriptase Kit (RL201-01, Vazyme) was used to synthesize cDNA with 2 µg extracted RNA. Subsequently, Taq Pro Universal SYBR qPCR Master Mix (Q712-02, Vazyme) was used to measure the expression of CUX1 and GAPDH mRNA (serving as an internal control) in blood or cell samples. The relative expression of CUX1 mRNA was calculated using the 2^−ΔΔCt^ method. Primer sequences are shown in Table 1, and all primers were synthesized by Sangon Biotech (Shanghai) Co., Ltd.

**Table 1:** Primer sequences

Gene name	Primer Sequences (5’ to 3’)
CUX1	F: TCCGTAGCATCCAAGGCAGACA
	R: CTTCATCAGAGCCAGTCTCCGA
GAPDH	F: GTCTCCTCTGACTTCAACAGCG
	R: ACCACCCTGTTGCTGTAGCCAA

### Cell proliferation analysis with CCK8 viability assay

A Cell Counting Kit-8 (CCK-8) kit (A311-01, Vazyme) was used to assess the effect of CUX1 on the proliferation of HK-2 cells induced by CM. First, the treated HK-2 cells in each group were seeded into a 96-well plate at 5 × 10^3^ cells/well and cultured until they completely adhered to the wall. At 0 and 24 h after adhesion, detection of cell proliferation was performed. Specifically, 10 µL of CCK-8 solution was added to cells in each cell with continued incubation for 2 h. Subsequently, the absorbance was measured at the wavelength of 450 nm using a Multiskan FC microplate photometer, and the cell viability was calculated according to the absorbance. Three independent experiments were performed.

### Analysis of apoptosis by Flow cytometry

The apoptosis rate of HK-2 cells in each group was determined using Annexin V-FITC cell apoptosis detection kits. Briefly, treated HK-2 cells were seeded into a 6-well plate at 5 × 10^5^ cells/well and cultured for 24 h. After washing with PBS, at least 1 × 10^6^ cells were collected and gently resuspended with 195 µL of Annexin V- fluorescein isothiocyanate isomer I (FITC)-binding solution. Afterward, 5 µL FITC-Annexin V and 10 µL propidium iodide were added to the cells and incubated for 30 min at ambient temperature in the dark. Finally, the apoptosis of HK-2 cells in each group was detected using a flow cytometry (BD FACSCanto II).

### LDH, MDA and cytokines kit detection

Commercial kits were used to check LDH leakage, interleukin (IL)-1β and tumor necrosis factor alpha (TNF-α) levels, and malondialdehyde (MDA) content in HK-2 cells to assess the effects of CUX1 in CM-induced HK-2 cells, Specifically, the culture supernatant of HK-2 cells in each treatment group was collected and then centrifuged at 4℃ and 12,000 rpm for 10 min to remove cell debris. Next, the leakage of LDH from HK-2 cells was tested with a LDH cytotoxicity assay kit (C0016, Beyotime, Shanghai, China). After addition of trypsin, HK-2 cell culture media FROM each group was collected. Subsequently, at least 1 × 10^7^ cells were lysed with an ultrasonic cell crusher and then centrifuged at 4℃ and 12,000 rpm for 10 min, and the cell debris was removed. An MDA content assay kit (S0131M, Beyotime), IL-1β detection kit (ab197742, Abcam, UK), and TNF-α detection kit (ab285312, Abcam) were used to detect the respective indicators in HK-2 cells, followed by calculation of the corresponding activity and content levels.

### Western blot analysis

HK-2 cells (at least 1 × 10^7^cells) were collected and added to RIPA lysis buffer (Beyotime, Shanghai, China). Subsequently, cells were ultrasonically disrupted for 3 min and centrifuged at 4 °C and 12,000 g for 10 min, and the supernatant was collected. The concentration of total protein extracted was determined using a bicinchoninic acid (BCA) protein assay kit (Beyotime, Shanghai, China). Next, 20 µg of total protein was mixed with 5× loading buffer and boiled for 10 min. After 12% sodium dodecyl sulfate-polyacrylamide gel electrophoresis, the protein was transferred to polyvinylidene fluoride membrane. The membrane was blocked in 5% skimmed milk powder for 2 h, and then incubated with primary antibodies overnight at 4℃ on a shaker. Primary antibodies used were: CUX1 antibody (1:1000; ab309139, Abcam, UK) phosphorylated phosphoinositide 3-kinase (p-PI3K, Tyr458) antibody, (1:1000; #17,366, Cell Signaling Technology, Danvers, MA, USA); PI3K antibody, (1:1000; #4249); phosphorylated Akt (p-AKT, Ser473) antibody (1:1000; #4060); AKT antibody (1:1000; #9271); and β-actin antibody (1:1000; ab6276, Abcam). Subsequently, goat anti-rabbit or mouse secondary antibody (1:3000; ab6721 and ab205719, respectively, Abcam, UK) was added for 1 h of incubation. The membrane was then immersed in enhanced chemiluminescence reagent (SuperSignal ECL, ThermoFisher), and immunoreactive bands were imaged using a gel-imaging system. ImageJ software was used to quantify the gray value of protein bands. Finally, the relative protein level of target protein was evaluated with β-actin as an internal control.

### Statistical analysis

All experimental results in this study involved at least three independent experiments. Data are expressed by mean ± standard deviation (SD) and visualized with GraphPad Prism 9.0 software. SPSS 22.0 software was used for statistical analysis. The difference between two groups was compared by unpaired (independent) parametric t-test, and the difference among multiple groups was compared by one-way analysis of variance. *P* < 0.05 indicated a significant difference.

## Results

### Evaluating the CUX1 levels in the blood of patients with CIAKI and in the HK-2 cell model of CM-induced AKI

**Fig. 1:**
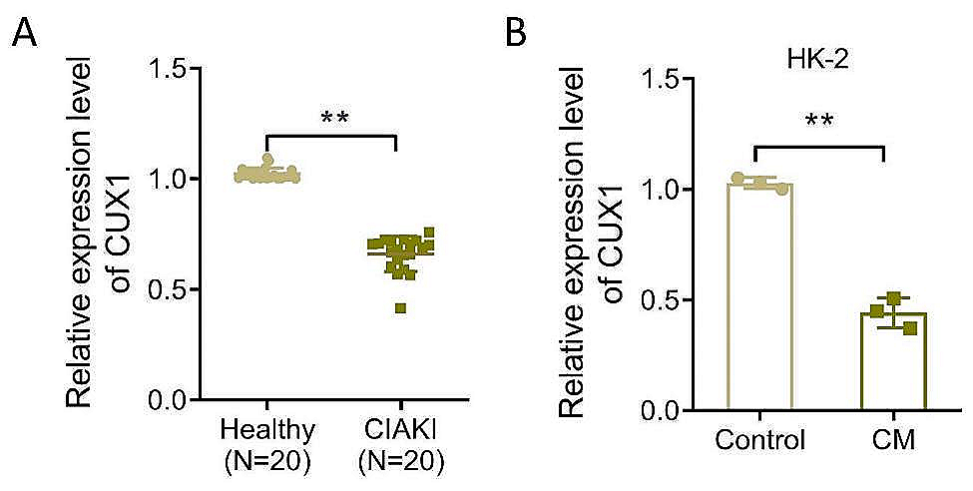
Levels of CUX1 mRNA in blood of patients with CIAKI and in the HK-2 cell model. (**A**–**B**), RT-qPCR measurement of levels of CUX1 mRNA in the blood of healthy volunteers and in patients with CIAKI (A, *n* = 20) and in HK-2 cells cultured normally and or induced with contrast media (B, *n* = 3). **P < 0.01. CIAKI, Contrast-induced acute kidney injury; RT-qPCR, Real-time quantitative polymerase chain reaction; CUX1, CUT-like homeobox 1; HK-2, human kidney 2

To clarify the role of CUX1 in patients with CIAKI, the expression level of CUX1 in clinical blood samples was first detected. The results showed that the relative level of CUX1 mRNA in the blood of patients with CIAKI was significantly lower than that in the blood of healthy volunteers (Fig. 1A, *P* < 0.01). Interestingly, the expression level of CUX1 was significantly decreased by nearly 2.32-fold compared with that in the control group in the CIAKI model of HK-2 cells induced by CM in vitro (Fig. 1B, *P* < 0.01). Overall, the low CUX1 expression in CIAKI samples may be related to CM-induced kidney injury.

### CUX1 promotes cell proliferation and inhibits cell apoptosis in CM-induced HK-2 cells and regulate the PI3K/AKT-signaling pathway

**Fig. 2:**
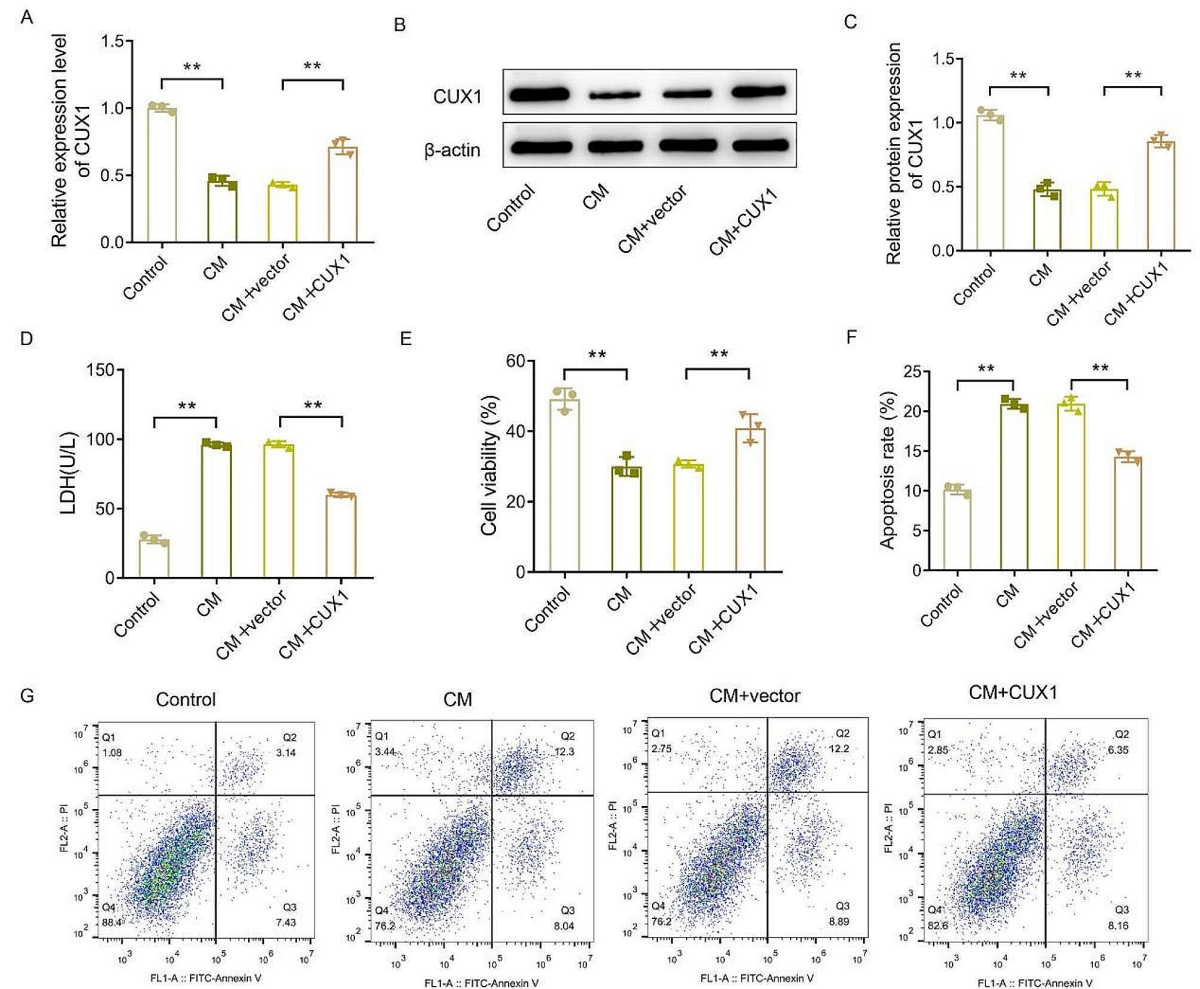
Effect of CUX1 on proliferation and apoptosis of HK-2 cells induced by CM. (**A**–**C**), RT-qPCR (**A**) and western blot (**B**–**C**) to determine the relative expression level of CUX1 mRNA and protein levels in HK-2 cells treated with CM and/or CUX1 overexpression vector; (**D**), Evaluation of LDH leakage in the culture supernatant of HK-2 cells treated with CM and/or CUX1 overexpression vector; E–G, CCK-8 (**E**) and flow cytometry (**F**–**G**) to assess the effects of CM and/or CUX1 overexpression vector on the proliferation and apoptosis of HK-2 cells. *N* = 3, ** *P* < 0.01. CUX1, CUT-like homeobox 1; CM, contrast medium; HK2, human kidney 2; LDH, lactate dehydrogenase; CCK-8, Cell Counting Kit-8

The low osmolar nonionic monomer iohexol was used to induce CIAKI. To clarify the effect of CUX1 on the proliferation and apoptosis of CM-induced HK-2 cells, CUX1 was overexpressed by transfection with a CUX1-overexpression plasmid in a HK-2–cell model induced by CM. RT-qPCR and western blot analyses showed that the relative mRNA and protein levels of CUX1 in HK-2 cells were significantly decreased after CM treatment (Fig. 2A–C, *P* < 0.01), which confirmed the RT-qPCR results. Moreover, relative to that in cells transfected with overexpression vector, the expression level of CUX1 was notably increased in CM-induced HK-2 cells transfected with CUX1 overexpression plasmid (Fig. 2A–C, *P* < 0.01). The LDH leakage assay showed that CM treatment significantly increased the LDH activity in the supernatant of HK-2 cells, whereas the overexpression of CUX1 could markedly reduce the LDH activity in CM-induced HK-2 cells (Fig. 2D, *P* < 0.01). Therefore, HK-2 cell damage was induced by CM while the overexpression of CUX1 could alleviate the damage to HK-2 cells caused by this. In addition, the functional results for proliferation and apoptosis showed that the proliferation level of HK-2 cells in the CM group was significantly lower than that in the control group, whereas the apoptosis level was markedly increased (Fig. 2E–G, *P* < 0.01). However, in contrast to the CM + vector group, the proliferation of HK-2 cells was notably promoted while apoptosis was inhibited in the CM + CUX1 group (Fig. 2E–G, *P* < 0.01). Collectively, up-regulation of CUX1 could alleviate the damage to the proliferation and apoptosis of HK-2 cells induced by CM.

### CUX1 alleviates the inflammatory response and oxidative stress induced in HK-2 cells by CM

**Fig. 3:**
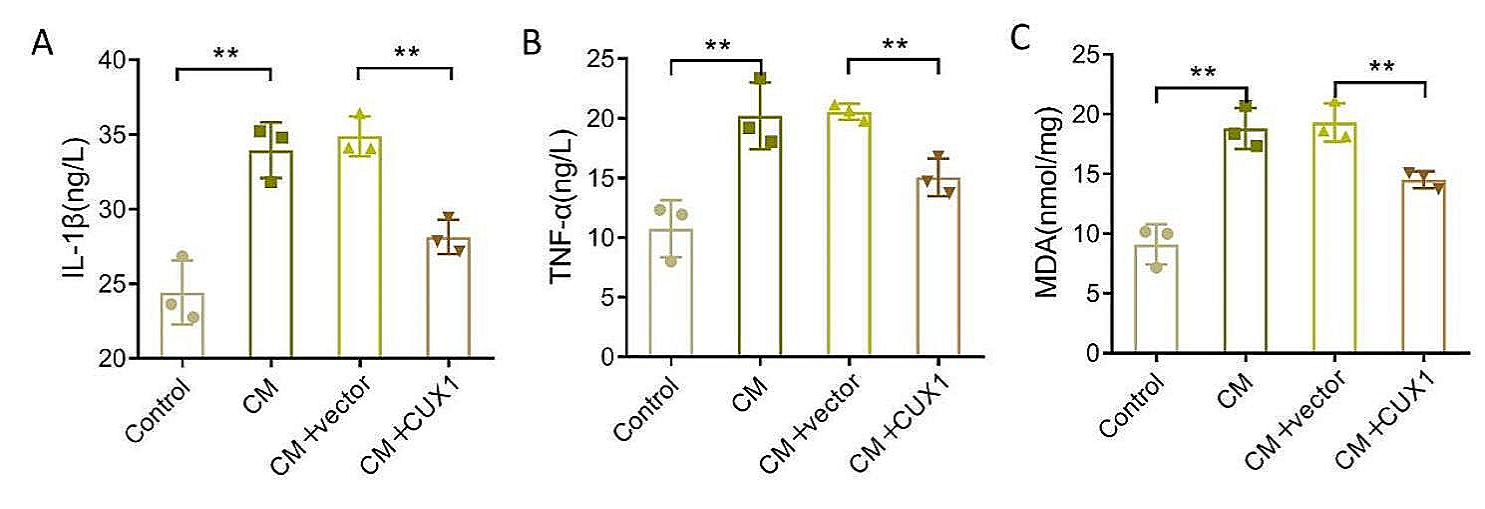
Influence of CUX1 on inflammatory response and oxidative stress in HK-2 cells induced by CM. (**A**-**C**), Evaluation of the levels of IL-1β, TNF-α, and MDA in HK-2 cells treated with CM and/or CUX1 overexpression vector. *N* = 3, ** *P* < 0.01. CUX1, CUT-like homeobox 1; CM, contrast medium; HK-2, human kidney 2; MDA, malondialdehyde; IL, interleukin; TNF-α, tumor necrosis factor alpha

To explore the effect of CUX1 on the inflammatory response and oxidative stress of HK-2 cells induced by CM, we examined the level of inflammatory factors IL-1β and TNF-α and that of the oxidative stress indicator MDA in HK-2 cells treated with CM and/or CUX1 overexpression vector. Briefly, in comparison with the control group, the levels of IL-1β, TNF-α, and MDA in HK-2 cells in the CM group were markedly upregulated, whereas in HK-2 cells in the CM + CUX1 group, these were much lower than in the CM + vector group (Fig. 3A–C, *P* < 0.01). Overall, CM-induced an inflammatory response and oxidative stress in HK-2 cells, whereas upregulation of CUX1 reduced these effects in CM-induced HK-2 cells.

### CUX1 increases the activity of the PI3K/AKT-signaling pathway in CM-induced HK-2 cells

**Fig. 4:**
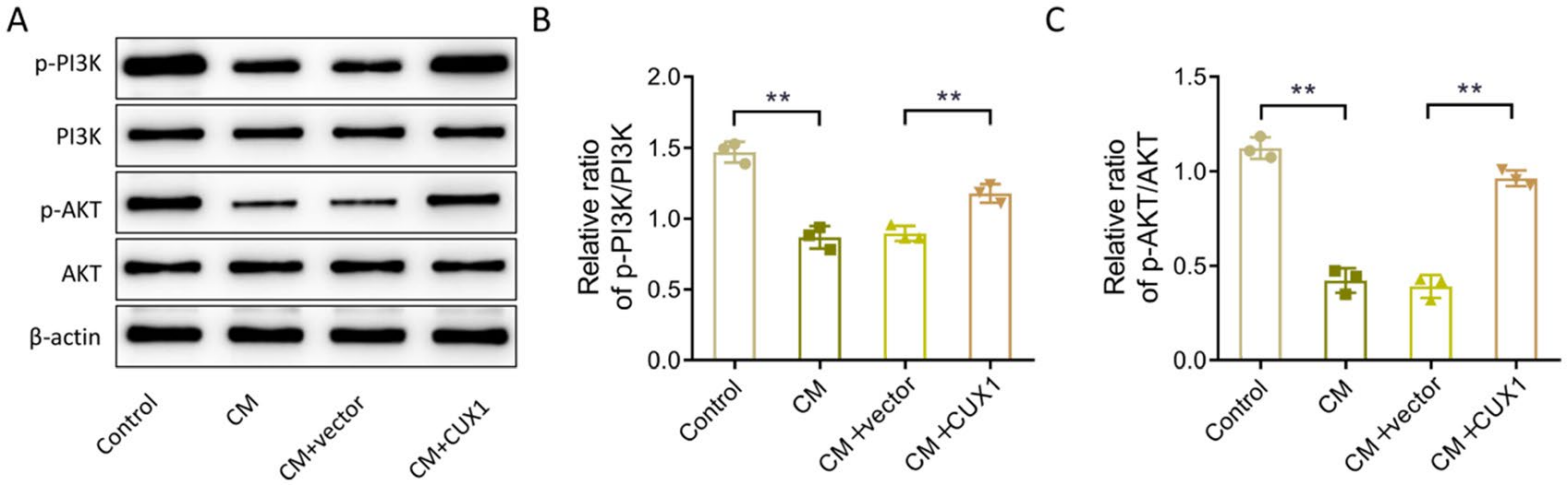
Effect of CUX1 on the activity of the PI3K/AKT-signaling pathway in HK-2 cells induced by CM. (**A**-**C**), Western blot evaluation of expression of PI3K/AKT-signaling pathway-related proteins (p-PI3K, PI3K, p-AKT, and AKT) in HK-2 cells treated with CM and/or CUX1 over-expression vector. *N* = 3, ** *P* < 0.01. p-PI3K, phosphorylated phosphoinositide 3-kinase; p-AKT, phosphorylated Akt; HK-2, human kidney 2; CM, contrast medium; CUX1, CUT-like homeobox 1

To explore the molecular mechanism of CUX1 in alleviating the CM-induced injury in HK-2 cells, western blotting was used to assess the effects of CUX1 overexpression vector and/or CM on the activity of PI3K/AKT-signaling pathway–related proteins (p-PI3K, PI3K, p-AKT, and AKT) in HK-2 cells. Compared with levels in the control group, the protein levels of p-PI3K and p-AKT and the ratios of p-PI3K/PI3K and p-AKT/AKT in the CM group were notably decreased. However, upregulating the expression of CUX1 significantly increased the levels of the above proteins and ratios (Fig. 4A–C, *P* < 0.01). Collectively, CM treatment significantly inhibited the activity of PI3K/AKT signaling pathway, while up-regulating CUX1 could activate the PI3K/AKT signaling pathway activity in HK-2 cells induced by CM.

## Discussion

CIAKI was first reported approximately 50 years ago [20]. Currently, there are millions of CIAKI cases, and a report has pointed out that CIAKI is considered to be the third cause of hospital-acquired AKI in hospitals in the United States [21]. In the United States alone, the demand for CM reached 30 million doses annually, indicating a considerable threat to the health of patients [22]. Although various biochemical markers have been discovered besides SCr, a reliable marker for timely diagnosis of CIAKI has yet to be identified [23, 24]. To find a more suitable biomarker, we evaluated the blood of patients with CIAKI and discovered that the expression of CUX1 was significantly decreased in these blood samples, suggesting that CUX1 may be a potential biomarker of CIAKI.

Early studies have shown that CI-AKI-induced cytotoxicity, leading to cellular dysfunction and apoptosis, occurs primarily by disrupting the function of renal tubular cells [25]. Clinically, iohexol is a common CM [26]. To determine effect of CM on the expression of CUX1 in patients, 200 mg/mL iohexol was used to induce renal tubular epithelial cell (HK-2 cells) for 6 h in vitro to construct a CIAKI model. We also found that CUX1 was highly expressed in HK-2 cells treated with iohexol. Combined with the high expression of CUX1 in the blood of CIAKI patients, it could suggest that the role of CUX1 deserves to be further explored. Moreover, CM treatment significantly reduced the proliferation level of HK-2 cells while notably increased the levels of apoptosis, inflammatory factors and oxidative stress-related substances. LDH is a lactate dehydrogenase, which is widely expressed in cells of different tissues, and it is the highest in the kidneys [27]. Studies reported that CIAKI could increase LDH activity [28]. Similar to the above results, in our study, CM treatment also increased LDH activity in the supernatant of HK-2 cells. However, overexpression of CUX1 significantly reduced LDH activity in HK-2 cells induced by CM. This result indicates that overexpression of CUX1 alleviates CIAKI, further increased the credibility of reduced CUX1 expression and enhanced kidney cell damage under CM stress. These findings agree with a previous result where CM treatment inhibited proliferation, promoted apoptosis, and encouraged inflammatory responses [19].

The expression level of CUX1 was significantly decreased in CM-induced HK-2 cells and blood samples of patients with CIAKI, further confirming the unbalanced expression of CUX1 in patients with CIAKI. Nevertheless, upregulating the expression of CUX1 could significantly increase the proliferation of HK-2 cells induced by CM and inhibit the apoptosis. The function of CUX1 in maintaining the normal proliferation of cells is reflected in the development of various tumors. Griesmann et al. considered that CUX1 was an important mediator for the proliferation and antiapoptosis of pancreatic cancer cells [26]. However, the functions of CUX1 in inhibiting cell proliferation and promoting cell apoptosis need to be taken into consideration. For example, CUX1 is a haploinsufficient tumor suppressor gene on chromosome 7 in acute myeloid leukemia [29]. Thus, CUX1 has a dual effect in regulating cell proliferation or apoptosis. As a transcription factor, CUX1 has a variety of subunits that have unique DNA binding abilities and transcription activities [30], which increase the complexity of how CUX1 functions. However, we consider that the downregulation of CUX1 expression can decrease the proliferation of HK-2 cells and increase their apoptosis.

CM could decrease blood flow, prolong CM excretion time, and ultimately lead to renal ischemia, hypoxia, increased oxygen free radicals, and kidney injury [31]. MDA is a metabolic product in living organisms. Under oxidative stress, intracellular lipids are oxidized, and produced a large amount of MDA, ultimately leading to an increase in the degree of oxidative stress. Studies reported that CIAKI could increase MDA activity [28, 32]. Similar to the results, in our study, CM treatment also increased the MDA activity of HK-2, however, overexpression of CUX1 significantly reduced the MDA activity in HK-2 cells induced by CM. This result indicates that overexpression of CUX1 alleviates the oxidative stress response of CIAKI. Ramdzan et al. demonstrated that CUX1 could repair oxidative DNA damage induced by a high level of reactive oxygen species (ROS) [33]. Additionally, CUX1 can significantly inhibit the activity of DNA damage–related pathways of ATM or p53 signals to resist the antiproliferation effect caused by ROS-induced DNA damage in CUX1-dependent cancer cells [34]. Thus, CUX1 has a considerable antioxidative stress effect. Herein, CM increased the level of oxidative stress in HK-2 cells, which decreased significantly once the expression of CUX1 increased. Therefore, the low expression of CUX1 may be the cause of oxidative damage of cells in CIAKI. IL-1β and TNF-α are two proinflammatory cytokines produced by M1 macrophages. A previous study showed that CM agents such as iodohexol can induce renal inflammation and upregulate the levels of inflammatory factors such as IL-1β, IL-6, and TNF-α [35, 36]. CUX1 was previously considered as an effector of TNF-α and a transcription factor that initiated the expression of IL-17 [37]. Nevertheless, these studies were performed using mouse stromal fibroblasts or intestinal epithelial cells, and none of them observed the effect of CUX1 on the production of inflammatory factors in HK-2 cells. In the current study, CUX1 reduced the level of IL-1β and TNF-α in cells. In view of the transcription function of CUX1 replication, CUX1 possibly has a two-way regulation of inflammatory response. Hence, we believe that the decrease in CUX1 expression in CIAKI is also the cause of excessive inflammatory response.

Based on previous studies, the PI3K/AKT signaling pathway is the main regulatory pathway of cell proliferation and apoptosis [38]. In AKI, activation of the PI3K/AKT pathway can reduce apoptosis and pyroptosis of HK-2 cells mediated by oxidative stress [39]. Similarly, in CIAKI, transfection of active AKT into CM-induced HK-2 cells can significantly increase the vitality of the cell model [40]. Moreover, a great many studies have claimed that the protection of HK-2 cells is mainly achieved by activating the PI3K/AKT pathway to improve CIAKI. For instance, Xie et al. thought that relaxin could attenuate CM-induced human proximal tubular epithelial cell apoptosis by activating the PI3K/AKT signaling pathway in vitro [41]. Liu et al. found that salvianolic acid B could prevent iodinated CM-induced acute renal injury in rats by activating the PI3K/AKT pathway [42]. In our study, we found that the activity of PI3K/AKT in CM-induced HK-2 cells decreased significantly and that the phosphorylation level of PI3K and AKT increased notably after upregulating CUX1 expression. Although the mechanism of CIAKI is not fully understood, most of studies believe that CM mainly activates renal tubular epithelial cell apoptosis through direct CM toxicity, vasoconstriction and oxidative stress, thereby leading to kidney function loss and acute kidney injury [7]. Studies reported that Baihe Gujin Pill prevented CIAKI by inhibiting apoptosis, oxidative stress, and inflammation via regulating PI3K/AKT and NF-κB/MAPK pathways [40], D-4 F ameliorates CM-induced oxidative injuries in endothelial cells via the AMPK/PKC Pathway [43]. In summary, more experiments are needed to further verify the molecular mechanism of CUX1 on CIAKI.

This study has some limitations: (1) Only cell model in vitro was conducted, and in vivo animal experiments need to be further implemented to explore the role and mechanism of CUX1 in CM-induced AMI. (2) Regarding how CUX1 affects the PI3K/AKT pathway and whether it plays a direct or indirect role in authorship, this needs to be further explored. (3) We only detected the activity of the PI3K/AKT-signaling pathway. Currently, it is unclear whether CUX1 plays a role in alleviating CM-induced AMI through other signaling pathways, and further analysis is required.

## Conclusion

To sum up, CUX1 expresses lowly in the blood of CIAKI patients and CM-induced HK-2 cells. Over-expression of CUX1 in CM-induced HK2 cells can significantly promote cell proliferation, inhibit cell apoptosis, reduce inflammatory response and oxidative stress, thereby alleviating CM-induced HK-2 damage. Based on further mechanism exploration, the effect of CUX1 above may be associated with activation of the PI3K/AKT signaling pathway. Consequently, CUX1 may be a potential biomarker for the treatment of CIAKI and requires further investigation.

### Electronic supplementary material

Below is the link to the electronic supplementary material.

Supplementary Material 1

## Data Availability

The data used to support the findings of this study are available from the corresponding author upon request.
