# Supplementary material for: CUX1 attenuates the apoptosis of renal tubular epithelial cells induced by contrast media through activating the PI3K/AKT signaling pathway
Source: BMC Nephrol. 2024 Jun 7;25:192. doi: 10.1186/s12882-024-03625-8 (PMC11162042; doi:10.1186/s12882-024-03625-8)
Supplement: Supplementary file 1 — Supplementary Material 1 [file 12882_2024_3625_MOESM1_ESM.pdf]

Figure 2B

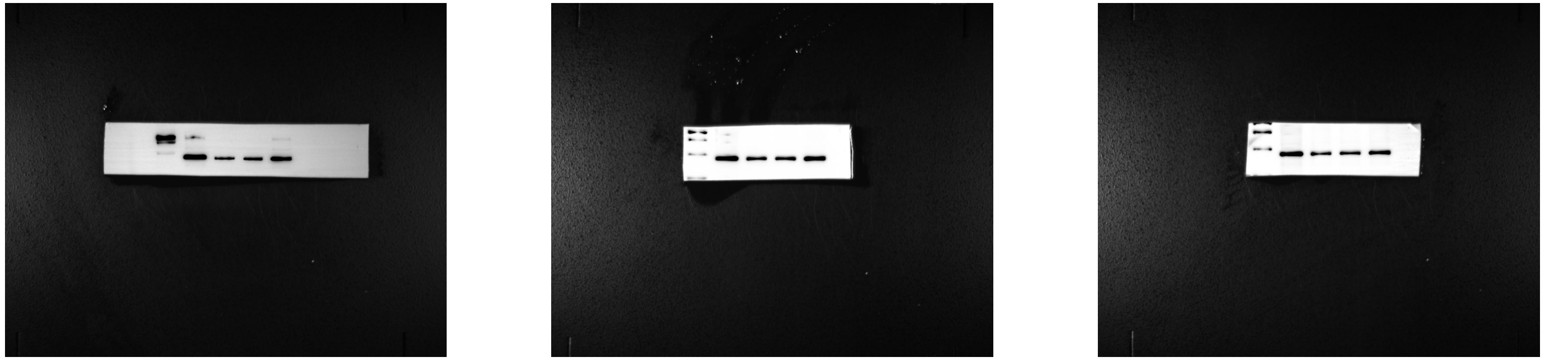

The original Western Blot images of CUX1 in Figure 2. From left to right: Control, CM, CM + vector and CM + CUX1.

Figure 2B

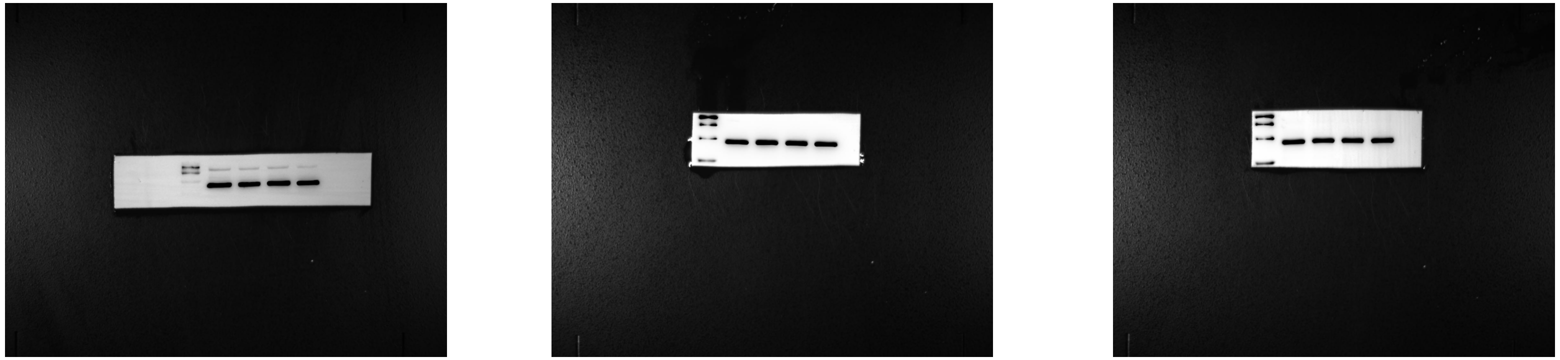

The original Western Blot images of β-actin in Figure 2. From left to right: Control, CM, CM + vector and CM + CUX1.

Figure 4A

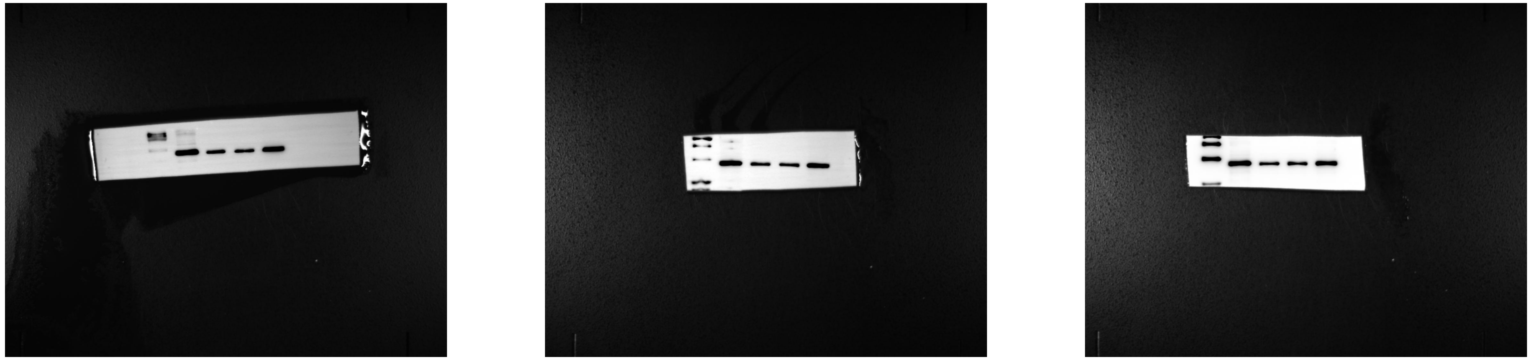

The original Western Blot images of p-PI3K in Figure 4. From left to right: Control, CM, CM + vector and CM + CUX1.

Figure 4A

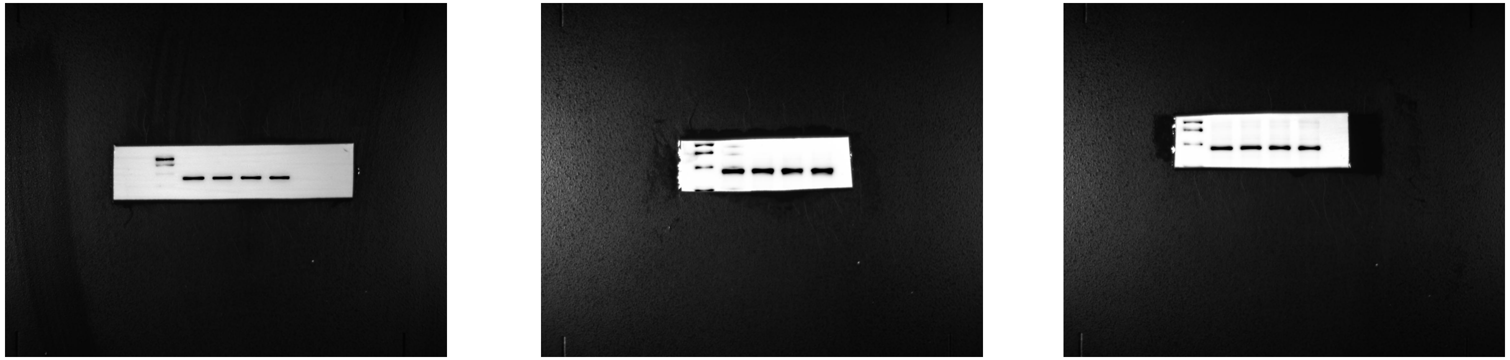

The original Western Blot images of PI3K in Figure 4. From left to right: Control, CM, CM + vector and CM + CUX1.

Figure 4A

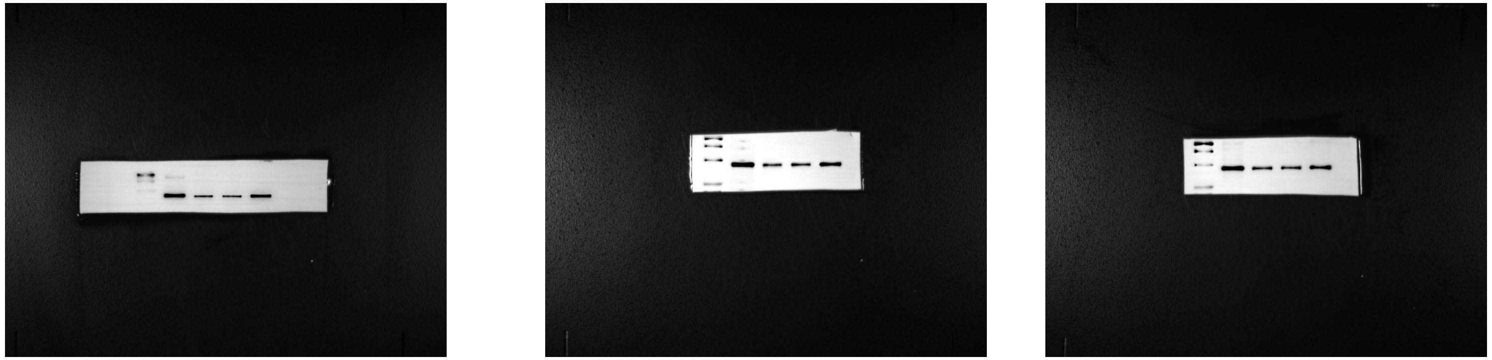

The original Western Blot images of p-AKT in Figure 4. From left to right: Control, CM, CM + vector and CM + CUX1.

Figure 4A

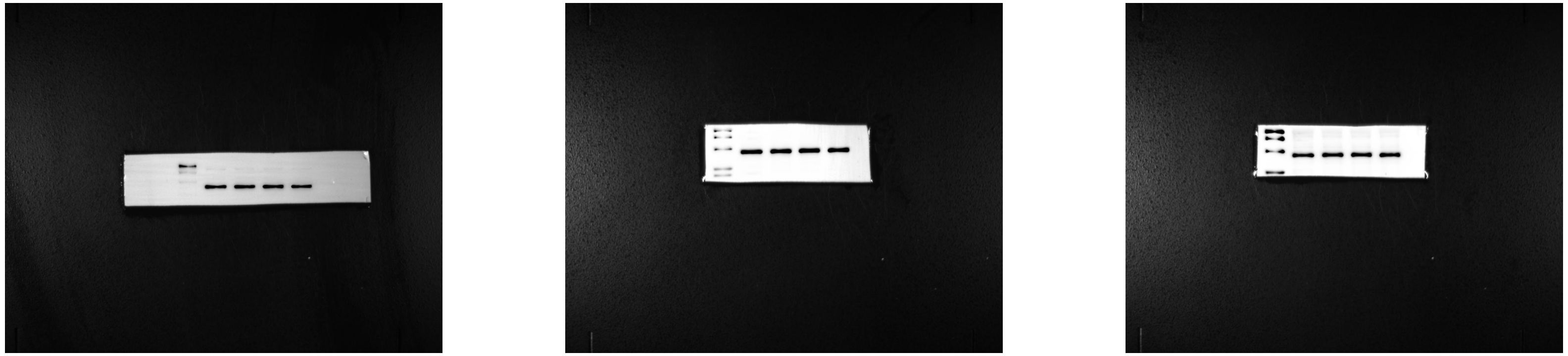

The original Western Blot images of AKT in Figure 4. From left to right: Control, CM, CM + vector and CM + CUX1.

Figure 4A

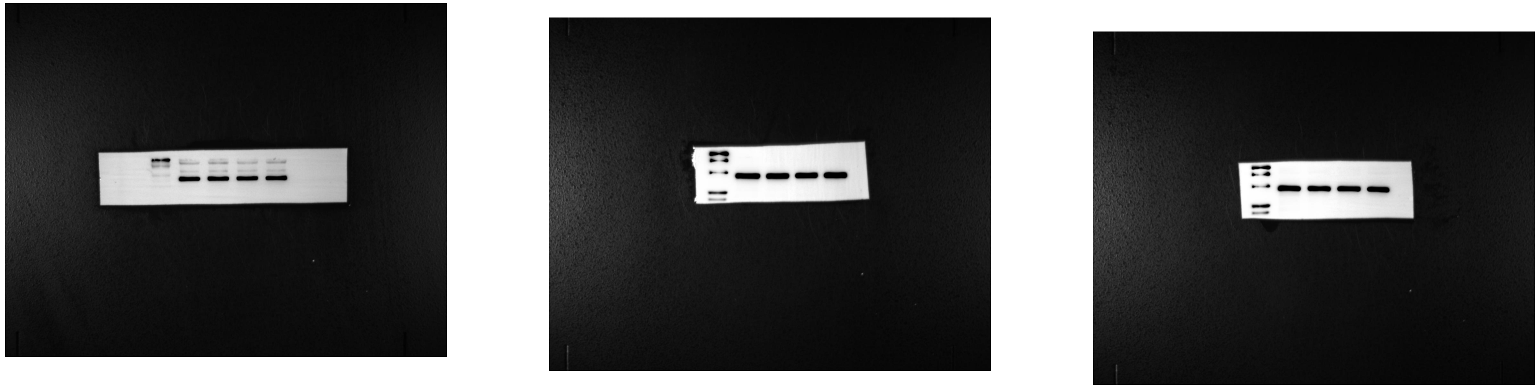

The original Western Blot images of β-actin in Figure 4. From left to right: Control, CM, CM + vector and CM + CUX1.
